# Management of persistent postoperative wound leakage after total hip and knee arthroplasty: a regional perspective in the north west of England

**DOI:** 10.1308/rcsann.2025.0002

**Published:** 2025-04-03

**Authors:** M Choi, A Wheelton, T Naylor, North West Orthopaedic Research Collaborative (NWORC)

**Affiliations:** ^1^ Manchester University NHS Foundation Trust, UK; ^2^ Northern Care Alliance NHS Foundation Trust, UK; ^3^ East Lancashire Hospitals NHS Trust, UK

**Keywords:** Wound leakage, Wound drainage, Prosthetic joint infection, Arthroplasty

## Abstract

**Introduction:**

There is a well-documented association between persistent wound drainage and the development of prosthetic joint infections in total hip (THA) and total knee arthroplasty (TKA). Despite this, there are no national clinical guidelines in the United Kingdom on the diagnosis or management of postoperative wound drainage. We aimed to evaluate what variability exists within clinical practice in the recognition and treatment of persistent wound leakage following THA and TKA.

**Methods:**

An anonymous online survey consisting of 12 multiple-choice questions was distributed among hip and knee arthroplasty consultants in the north west of England. Topics covered in the questionnaire included definition, diagnosis, classification, timing and treatment of persistent wound drainage.

**Results:**

Twelve orthopaedic centres across the region participated in data collection. A total of 65 consultants completed the questionnaire. Some 45% of respondents used a definition of persistent wound leakage after arthroplasty, which ranged from drainage beyond 48h to that lasting more than 2 weeks. Only 54% of consultants reported having a monitoring system in place for patients with persistent wound drainage after discharge from hospital. There was wide variation in the preferred timing of initiating both non-operative and surgical management of wound leakage, as well as different treatment modalities used. Most respondents rated C-reactive protein as the most useful serological marker in aiding decision making.

**Conclusion:**

The results demonstrate a lack of concurrence in the recognition and management of postoperative wound drainage. Formal national clinical guidelines are necessary to standardise practice.

## Introduction

One of the numerous potential complications facing arthroplasty surgeons is postoperative wound leakage. In addition to contributing to prolonged hospital stays, patient distress and impeding mobilisation, persistent wound drainage carries the potential to impact long-term patient outcomes.^1^ It has been shown that total knee arthroplasty (TKA) patients readmitted with wound complications, including from non-infectious causes, reported lower mean functional scores, with 40% more patients experiencing persistent pain at 2 years postoperatively compared with the control group.^2^ There is also a well-documented association between persistent wound drainage and the development of prosthetic joint infection (PJI) in total hip arthroplasty (THA) and TKA, with one study reporting that 88% of PJI patients experienced postoperative wound leakage compared with 36% of patients with no subsequent infection.^3,4,^^5^ It has also been stipulated that patients who have developed PJI following a hip or knee arthroplasty were 16.8 times more likely to have experienced persistent wound leakage than those without PJI.^6^ PJI is known to place a tremendous burden on patients and healthcare costs associated with its operative management in the form of revision surgery. This is due to factors such as prolonged procedure duration, need for multiple operations, increased blood loss, and increased use of bone allograft, as well as a higher patient morbidity and mortality risk.^7^

The International Consensus Meeting on PJI in 2013 defined persistent wound leakage as a greater than 2 × 2cm drainage in the wound dressing beyond 72h after the index procedure. There was strong consensus at the time that patients with persistent discharge for 5–7 postoperative days should undergo surgical management without delay.^8^ There were no updates on this topic in subsequent International Consensus Meetings on Musculoskeletal Infection in 2018^9^ and 2023.^10^

Getting It Right First Time (GIRFT), a United Kingdom (UK) programme designed to improve patient care, recognises wound ooze as a possible indicator of a deep postoperative infection, and that oozing should cease by 72h after surgery.^11,^^12^ Despite these recommendations, there are no formal national clinical guidelines in place in the UK on the diagnosis or management of postoperative wound drainage. The aim of this study was to evaluate what variability, if any, exists within clinical practice in the north west of England in the recognition and treatment of persistent wound leakage following primary THA and TKA.

## Methods

A questionnaire on the management of persistent wound leakage in the Netherlands published by Wagenaar *et al* was used as a template and adapted to create an online survey tailored to UK practice.^13^ This consisted of 12 multiple-choice questions, with 10 further optional free-text answers (Appendix 1 – available online). Topics covered in the questionnaire included the definition, diagnosis, classification, timing and treatment of persistent wound drainage. Responses were collected anonymously from hip and knee arthroplasty consultant surgeons, between 14 April and 6 October 2023.

The questionnaire was created using the Google Forms interface. It was distributed among orthopaedic centres in the north west of England, covering Greater Manchester, Lancashire and Cumbria regions.

Microsoft Excel was used for statistical analysis with comparison of percentage proportions for each question. Because of the observational nature of the study without patient involvement, it was determined that formal ethical approval was not required.

To the authors’ knowledge, there is no standardised care pathway for lower limb arthroplasty patients across the north west of England, although all departments follow the National Health Service (NHS) Enhanced Recovery Programme^14^ and the Elective Hip and Knee Replacement recommendations from GIRFT.^15^ A number of orthopaedic centres have day-case protocols for select hip and knee arthroplasty patients.

## Results

Twelve orthopaedic departments, of a possible 13 in the region (92%), participated in data collection, with a total of 65 responses recorded. A list of participating hospitals and their according response rates is available in Appendix 2 (available online). A range of clinical experience was recorded among the clinicians, with the largest proportion of 34% having between 10 and 20 years of consultant-level experience. An equal proportion, 20% each, reported between 5 and 10 years and more than 20 years of experience. A duration of experience between 0 and 5 years was reported by 26% of consultants.

### Definition of persistent wound drainage

**Figure 1:**
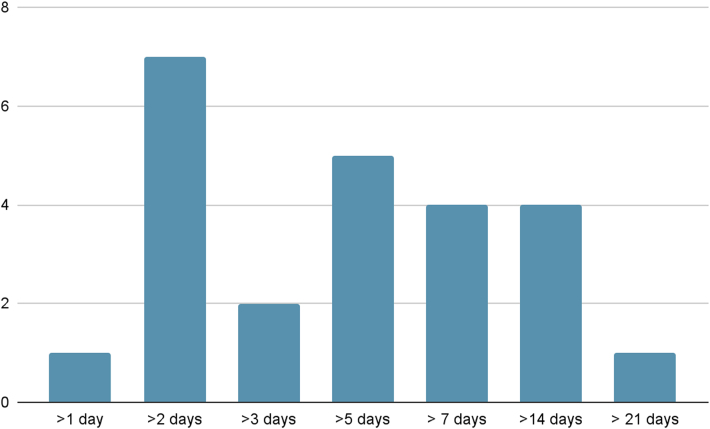
Definitions of persistent wound leakage

A definition of persistent wound leakage after arthroplasty was used by 29 (45%) respondents. Of this cohort, 25 surgeons (86%) utilised a temporal denotation; wound drainage lasting more than 2 days was the most popular definition used by seven respondents. Figure 1 gives the definitions used.

Other responses included “If/when leakage breaches the sides of an Opsite dressing”, “An area of >0.5–1cm diameter of strikethrough on dressing”, “Leaky wound with surrounding erythema and boggy swelling” and “Change of dressing required daily”.

### Classification system, clinical protocol and postdischarge monitoring

Only two (3%) respondents reported using a classification system of persistent wound leakage. However, when asked to comment with further detail on the system used, no relevant responses were provided. Surgeons were asked to comment on whether they follow a clinical protocol for patients with wound drainage; 25 (38%) respondents did not follow a protocol. A smaller proportion of 22 (34%) consultants followed a protocol in all cases, and 18 (28%) did in select cases.

Almost half of respondents (*n* = 32, 49%) did not discharge patients from hospital with active wound leakage, with a further 24 (37%) rarely doing so, and 9 (14%) discharged these patients most of the time. Thirty-five consultants (54%) had a monitoring system for active wound leakage after hospital discharge. In most cases this consisted of early review in clinic within 1–2 weeks or nurse-led follow-up. The remaining 46% reported no monitoring system in place for patients discharged from hospital.

### Non-operative management of persistent wound drainage

**Figure 2:**
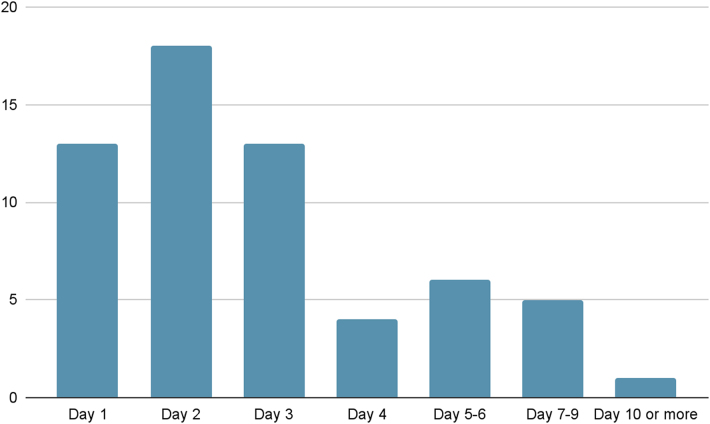
Timing of non-surgical management following persistent wound drainage

Surgeons were asked to comment on when they would commence non-surgical treatment following persistent wound leakage. A variety of responses were provided, with duration of drainage ranging from 1 day, to that lasting more than 10 days (Figure 2). The largest group of consultants, 18 (28%), elected to start non-operative management on day 2. There were four ‘other’ responses recorded. Two respondents commented that the decision is either case or leakage content-dependent, and one commented that they “would aspirate or take to theatre.” A further two clinicians commented that they did not understand the question.

**Figure 3:**
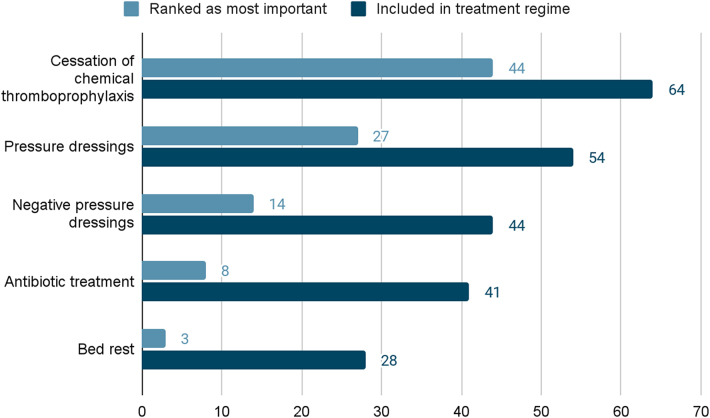
Non-surgical treatment modalities

Consultants were further questioned on what treatment modalities comprise their non-operative management regime. They were asked to select, in order of importance, from five treatment options, which included pressure dressings, antibiotics, negative pressure wound therapy, temporary cessation of chemical thromboprophylaxis and bed rest. Multiple selections were possible. The results are presented in Figure 3. Cessation of chemical thromboprophylaxis was the most popular choice, selected by 64 (99%) respondents, and also considered the most important measure by 44 (68%) surgeons. There was also an option to record ‘other’ free-text responses; these are listed in Table 1.

**Table 1:** Other responses included in non-surgical treatment modalities

Free-text responses recorded
Tranexamic acid
Investigations (bloods)
If TKR – restrict flexion. If THR – lie on contralateral side with pillow between legs
Check albumin, LFT, clotting screen. Turn patient on contralateral side to THR
Wound swab after 48h and splint; antibiotics if positive culture
Cessation of any other anticoagulants not related to the post op VTE prophylaxis
Brace for knee
If a knee, short-term splintage can be helpful
Clexane stopped for 72h and oral tranexamic acid 500mg tds prescribed for 72h. Mechanical VTE prophylaxis used (flowtron or equivalent)
Medication review
Limb elevation, drug review, cryocuff

LFT = liver function tests; THR = total hip replacement; TKR = total knee replacement; VTE = venous thromboembolism

### Operative management of persistent wound drainage

**Figure 4:**
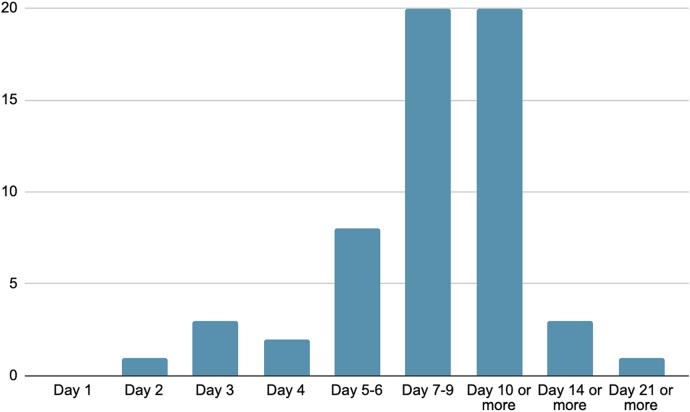
Timing of surgical management following persistent wound drainage

The questionnaire also sought responses on when consultants would commence surgical treatment for a persistently leaky wound. Results are presented in Figure 4, with a wide range of practice demonstrated. Consultants were evenly split on starting operative management on wound drainage going on for 7–9 days, and 10 or more days, with 20 (30%) responses recorded for each. There were seven ‘other’ responses and these are presented in Table 2.

**Table 2:** Other responses included in timing of surgical management

Free-text responses recorded
Impossible to say unless day that leakage becomes persistent is defined
Depends on contents of leakage
Will have to consider patient factors to decide individually
Case dependent
Too closed a question, depends on factors such as haematoma so nay between day 0 and beyond day 10
Depends on severity of leak
Case dependent

**Figure 5:**
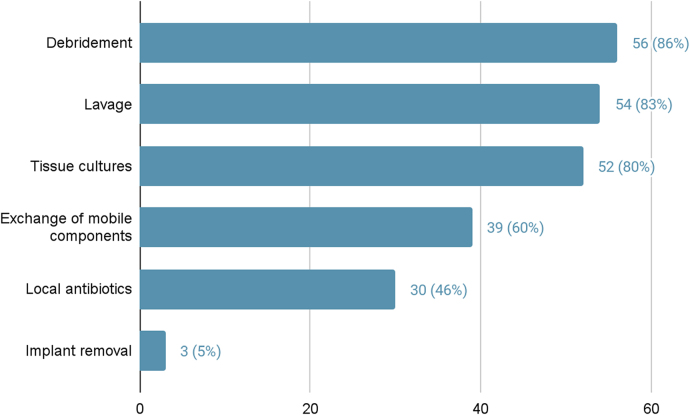
Surgical treatment modalities

Respondents were then asked to specify which treatment modalities they include in their operative management regime. They had the option to select from six choices – debridement, lavage, tissue cultures, exchange of mobile components, local antibiotics and implant removal – with multiple answers possible. The results are presented in Figure 5. It was also possible to opt for ‘other’ free-text responses, which are listed in Table 3. More than 80% of surgeons used a combination of debridement, lavage and tissue cultures.

**Table 3:** Other responses included in surgical treatment modalities

Free-text responses recorded
Case dependent, if superficial not connected to joint for washout, debridement plus primary closure. DAIR for deep collection
Full radical synovectomy
Depends on layer of debridement needed and whether it becomes a DAIR
Dependent on case
Case dependent? infection? depth
As per case need
Chemical debridement
Depends on clinical and intra-operative findings
Cement spacer
Any of above dependent on the case

DAIR = debridement, antibiotics and implant retention

A final question in this category concerned whether at the time of a surgical wound washout, the clinician would choose to proceed with a formal deep debridement and washout in the presence of a subcutaneous collection if the fascia was felt to be clinically intact. A response of ‘yes’ was recorded for 19 (29%) consultants, ‘no’ for 18 (28%), and 26 (40%) stated the decision was case dependent. Two ‘other’ responses were recorded, both of which involved aspirating the joint first to check for a deep collection.

### Serological markers

C-Reactive protein (CRP) was used by 43 (66%) of respondents in their decision-making process in the treatment of persistent wound leakage after arthroplasty, whereas 22 (34%) did not utilise this marker.

CRP was also the most important serological marker for 36 (55%) of consultants, followed by white cell count with 5 (8%) votes, and erythrocyte sedimentation rate with 3 (5%). Thirteen (20%) clinicians did not consider serological markers important. Other responses included platelets, albumin, clotting profile, overall trend and case-dependent approach. No details were shared regarding inflammatory marker level thresholds for prompting more aggressive surgical management.

### Associated clinical parameters

**Figure 6:**
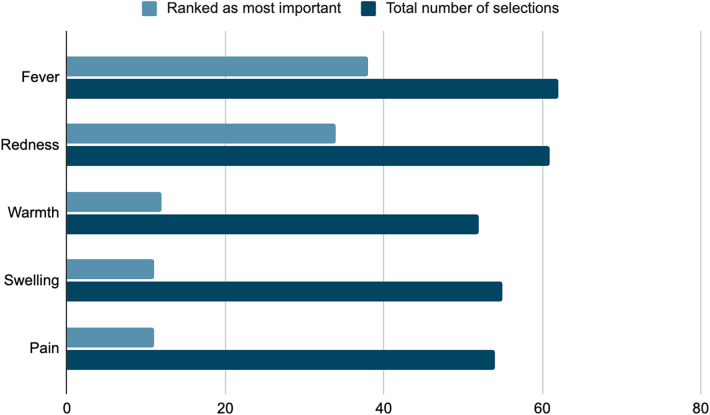
Associated clinical parameters

Surgeons were asked to select, in order of importance, which clinical parameters they used as part of their decision making in managing persistent wound drainage, out of redness, fever, pain, warmth and swelling. Multiple selections were possible. The results are illustrated in Figure 6. Fever and redness were the two most popular choices.

## Discussion

The questionnaire results demonstrate a lack of consensus among clinicians on the recognition and management of postoperative wound leakage, and considerable variability in practice. This is across an experience range of surgeons working in the capacity of arthroplasty consultants. The variability noted is consistent with a lack of formal evidence-based guidelines, as well as the challenging nature of managing this complication.

Although a definition of persistent wound drainage has been suggested by both the International Consensus Meeting group and GIRFT, most surgeons’ definitions did not align with the proposed 72h or more in duration.^8,^^11^ In most cases (73%), however, clinicians recognised wound leakage as persistent within the first 3 days of onset. Kremers *et al* postulated that 88% of PJI patients underwent early wound dressing changes in the first three postoperative days compared with 40% in the control group.^5^ Early recognition is therefore important in the subsequent attempts at mitigating the risks of PJI development.

A substantial proportion of surgeons, 46%, did not have a monitoring system for wound leakage after hospital discharge. According to a prospective multicentre study in the Netherlands, the strongest risk factors for PJI were any wound drainage in the second week, or moderate to heavy drainage in the second or third week post index procedure.^16^ The National Joint Registry (NJR) website states the average length stay after a total hip replacement is between 3 and 5 days; wound ooze that develops after hospital discharge and puts patients at significant risk of PJI development can be missed unless measures are put in place to actively monitor for this complication.^17^

Only 3% of respondents in the study claimed to use a classification system for persistent wound leakage, without providing further details on what system they used. It is recognised that there are no widely used clinical toolkits to aid in the classification of this complication. The only formal classification tool known to the authors is one proposed by Wagenaar *et al*, which stratifies wound leakage into four categories based on the amount of drainage present.^3^

There was marked variability in commencing and selecting both non-operative and surgical management measures. With multiple reports of an association between wound drainage and PJI, such as Saleh *et al* describing a 12.7 times increased risk of PJI with wound leakage lasting beyond 5 days, early intervention measures must be considered for this patient group.^4^ However, surgical intervention in particular needs to be balanced against the risks associated with undergoing a further operation, adding to the challenge in the management of this complication. The NJR, the largest orthopaedic registry in the world, records, monitors and reports on performance outcomes of arthroplasty surgery amongst orthopaedic departments and individual surgeons.^17^ A debridement, antibiotics and implant retention (DAIR) procedure, one surgical option in managing wound leakage, is recorded in the NJR as a revision operation, adding to a clinician’s overall revision rates. This may be a factor in deterring arthroplasty surgeons from undertaking early surgical intervention for this complication.

## Conclusion

This pilot study represents a sample of arthroplasty consultants’ clinical practice across the north west of England and will hopefully provide a framework for a national audit of practice. The degree of variability in recognising and managing persistent wound drainage highlights a need for the development of formal guidelines. The authors propose that until high-quality randomised trials addressing this issue are available and such guidelines are published, clinicians ought to adhere to the recommendations of GIRFT and the International Consensus Meeting on PJI, particularly in view of the plethora of evidence available linking persistent wound leakage to PJI risk.^8,^^11,12^ In addition, all orthopaedic departments should have wound drainage monitoring systems in place for arthroplasty patients following discharge from hospital, to help identify at-risk patients and implement appropriate management measures early. Further work needs to be undertaken to help guide operative and non-operative management of this complication; the authors look forward to the results of the LEAK study, a nationwide randomised controlled trial in the Netherlands aiming to identify the best treatment modality for persistent wound drainage following hip and knee arthroplasty, in anticipation.^18^
